# XPS Study on the Stability and Transformation of Hydrate and Carbonate Phases within MgO Systems

**DOI:** 10.3390/ma10010075

**Published:** 2017-01-18

**Authors:** Vanessa Rheinheimer, Cise Unluer, Jiawei Liu, Shaoqin Ruan, Jisheng Pan, Paulo J. M. Monteiro

**Affiliations:** 1 Berkeley Education Alliance for Research in Singapore, Singapore 138602, Singapore; 2 Department of Civil and Environmental Engineering, Nanyang Technical University, Singapore 639798, Singapore; ucise@ntu.edu.sg (C.U.); LI0015EI@e.ntu.edu.sg (J.L.); SRUAN001@e.ntu.edu.sg (S.R.); 3 Institute of Materials Research and Engineering, A*STAR (Agency for Science, Technology and Research), 2 Fusionopolis Way, Innovis, #08-03, Singapore 138634, Singapore; js-pan@imre.a-star.edu.sg; 4 Department of Civil and Environmental Engineering, University of California at Berkeley, Berkeley 94720, CA, USA; monteiro@berkeley.edu

**Keywords:** MgO, carbonation, hydration, XPS

## Abstract

MgO cements have great potential for carbon sequestration as they have the ability to carbonate and gain strength over time. The hydration of reactive MgO occurs at a similar rate as ordinary Portland cement (PC) and forms brucite (Mg(OH)_2_, magnesium hydroxide), which reacts with CO_2_ to form a range of hydrated magnesium carbonates (HMCs). However, the formation of HMCs within the MgO–CO_2_–H_2_O system depends on many factors, such as the temperature and CO_2_ concentration, among others, which play an important role in determining the rate and degree of carbonation, the type and stability of the produced HMCs and the associated strength development. It is critical to understand the stability and transformation pathway of HMCs, which are assessed here through the use of X-ray photoelectron spectroscopy (XPS). The effects of the CO_2_ concentration (in air or 10% CO_2_), exposure to high temperatures (up to 300 °C) and curing period (one or seven days) are reported. Observed changes in the binding energy (BE) indicate the formation of different components and the transformation of the hydrated carbonates from one form to another, which will influence the final performance of the carbonated blends.

## 1. Introduction

The high energy requirement and CO_2_ emissions associated with the production of Portland cement (PC), amounting to at least 5% of global anthropogenic emissions [1], has led to the development of alternative cementitious binding materials with lower environmental impact. One class of alternatives involves reactive magnesium oxide (MgO)-based binders, which have attracted significant attention in recent years [2]. MgO can be produced from readily available minerals (e.g., magnesite and dolomite) and waste brine obtained from desalination plants. Reactive MgO cements offer several advantages in terms of their technical and sustainable features when compared to traditional PC: in addition to the lower temperatures (i.e., 700 °C vs. 1450 °C for PC) required for their manufacturing, they have the ability to sequester significant quantities of CO_2_, resulting in carbon-neutral cements. The resulting construction materials can have high strengths, and they can be easily recycled, as their carbonation leads to the formation of carbonates from which MgO is predominantly obtained. In concrete mixes, reactive MgO can hydrate to form brucite (Mg(OH)_2_, magnesium hydroxide), which, when subjected to the right curing conditions, can react with CO_2_ and additional water to form a range of strength-providing hydrated magnesium carbonates (HMCs), as shown in Equations (1)–(4), with distinct water content, which define their structure:

MgO + H_2_O → Mg(OH)_2_ (brucite) (1)

Mg(OH)_2_ + CO_2_ + 2H_2_O → MgCO_3_∙3H_2_O (nesquehonite) (2)

5Mg(OH)_2_ + 4CO_2_ → 4MgCO_3_·Mg(OH)_2_·4H_2_O (hydromagnesite) (3)

5Mg(OH)_2_ + 4CO_2_ + H_2_O → 4MgCO_3_·Mg(OH)_2_·5H_2_O (dypingite) (4)

The HMCs can be categorized according to the number of Mg ions they contain within their structures, listed in Table 1. Magnesite and brucite are the most stable phases.

The type of HMC formed depends on the temperature and the CO_2_ concentration: temperature increases lead to the transformation of carbonates into less hydrated forms, whereas a change in the CO_2_ concentration results in the formation of different phases. An accelerated heating leads to the production of poorly-ordered carbonates, while gradual changes in the curing conditions result in the formation of crystalline intermediates. In addition to the CO_2_ concentration and temperature, other parameters, including the water activity and pH, influence the formation of different HMCs.

Lansfordite decomposes into nesquehonite at temperatures above 10 °C [3,6], since it has a lower stability field than nesquehonite [7]. At temperatures above 50 °C, nesquehonite, whose stability is influenced by the loss of water from the system, resulting in the reduction of the water activity, transforms into hydromagnesite [6]. Morgan et al. [8] recently reported that the stability of nesquehonite is enhanced in certain conditions (i.e., in the presence of CO_2_, under low temperature and in high humidity). Davies and Bubela [9] demonstrated that protohydromagnesite, a similar phase as dypingite, appears as an intermediate phase before hydromagnesite, which was also confirmed by Botha and Strydom [10] and Power [11]. Canterford et al. [12] stated that a range of intermediate phases between nesquehonite and hydromagnesite may form, according to Equation (5). In this equation, *x* can range between 4–8 and 11, where the resulting component would correspond to dypingite and giorgiosite when *x* is equal to 5 and 6, respectively:

(5) $$5\left({\mathrm{MgCO}}_{3}\cdot 3H_{2}O\right)\to {\mathrm{Mg}}_{5}{\left({\mathrm{CO}}_{3}\right)}_{4}{\left(\mathrm{OH}\right)}_{2}\cdot xH_{2}O+{\mathrm{CO}}_{2}+\left(15-\left(x-1\right)\right)H_{2}O$$

Hydromagnesite is transformed into magnesite under elevated temperatures (e.g., 126 °C) [13]. This transformation was found to be possible in both ambient and accelerated CO_2_ atmospheres, although the latter was observed to trigger the process. Based on the statement that the decomposition of HMCs depends on the loss of water [12], artinite can potentially decompose to pokrovskite, which contains less water in its structure. Overall, the transformation pathway of HMCs follows the trend shown in Equations (6) and (7):

(6) $$\begin{array}{ccccc} \mathrm{Lansfordite}\to & \mathrm{Nesquehonite}\to & \mathrm{Dypingite}\to & \mathrm{Hydromagnesite}\to & \mathrm{Magnesite}\\ {\mathrm{MgCO}}_{3}\cdot 5H_{2}O\to & {\mathrm{MgCO}}_{3}\cdot 3H_{2}O\to & {\mathrm{Mg}}_{5}{\left[\mathrm{OH}|{\left({\mathrm{CO}}_{3}\right)}_{2}\right]}_{2}\cdot 5H_{2}O\to & {\mathrm{Mg}}_{5}\left[{\left({\mathrm{CO}}_{3}\right)}_{4}{\left(\mathrm{OH}\right)}_{2}\right]\cdot 4H_{2}O\to & {\mathrm{MgCO}}_{3} \end{array}$$

(7) $$\begin{array}{cc} \mathrm{Artinite} & \to \mathrm{Pokrovskite}\\ {\mathrm{Mg}}_{2}\left[{\left(\mathrm{OH}\right)}_{2}|{\mathrm{CO}}_{3}\right]\cdot 3H_{2}O & \to {\mathrm{Mg}}_{2}\left[{\left(\mathrm{OH}\right)}_{2}|{\mathrm{CO}}_{3}\right] \end{array}$$

Chaka et al. [14] underline that the amount of water in the environment where the carbonation occurs strongly influences the transformation path of the HMCs and their stability, and they relate this to carbon storage and the sequestration potential.

The hydration and carbonation of MgO systems involves very complex stages that comprise several phases and involves the formation of secondary products. Thus, the durability and behavior of concrete during its service life strongly depends on its atomic structure. The transformation pathway of HMCs under different curing conditions, and its effect on the overall performance of construction products, has not been studied in detail until now. This plays a significant role in understanding the stability and determining the suitable end products of MgO cements. To facilitate a full understanding of the carbonation process, it is necessary to determine the factors that influence the transformation of these HMCs at different levels. This will not only enable the understanding and control of the properties that affect the formation of these strength-providing materials, but also enable the production of a range of sustainable construction products that rely on this system.

The chemical and physical properties of HMCs have been investigated by several researchers, who utilized analysis techniques, such as thermogravimetric analysis (TGA) and X-ray diffraction (XRD) to study individual phases [6,15,16,17,18]. While these methods reveal useful information about the crystalline structure and the thermal decomposition patterns of different phases, there are limitations to using the techniques to analyze systems in which different HMCs co-exist. TGA cannot be solely used to differentiate HMC phases from one another due to the similar decomposition patterns of these carbonates, which obscures the ability to assign phases for the dehydration, dehydroxylation, and decarbonation reactions. Furthermore, the overlapping crystalline peaks of different HMC phases also present a challenge in the identification of these HMCs via XRD. Alternative methods have to be considered with regards to investigating the phases and transformation pathways on HMCs, which provide information on the chemical state of each element.

X-ray photoelectron spectroscopy (XPS), which is employed in this investigation, enables the analysis of the chemical composition of the material surfaces by exposing them to a beam of X-rays and measuring the kinetic energy and the number of photoelectrons that escape from the surface. This provides spectra of all of the elements present within each system. Elemental identification is possible as the photoelectrons from each element have a group of specific binding energies, which is the difference between the photon energy and the kinetic energy of the measured photoelectrons. On the other hand, small changes in the chemical bonding environment cause changes in the photoelectron binding energy–chemical shift, providing chemical information. This study investigates the stability and transformation of magnesium carbonates under a wide range of conditions by studying the chemical changes over time of magnesium carbonates on the surface of the particles. As the curing conditions and binder characteristics determine the type of HMC formed and, consequently, the strength and carbon footprint of the structure, this work aims to investigate the HMCs formed under each curing condition using XPS.

## 2. Materials and Methods

The main cement binder used in this study was reactive MgO (commercial name, “calcined magnesite 92/200”) obtained from Richard Baker Harrison Ltd. (Staffordshire, UK). The chemical composition of MgO is presented in Table 2.

MgO and water were mixed in a bench-scale food mixer at a water/cement (w/c) ratio of 0.55. The different mixes were placed in 50-mm diameter metallic molds and pressed in a uniaxial press with a force of 5 kN. Afterwards, the samples were immediately demolded and placed in the relevant curing environments. The demolded samples were subjected to two curing environments for different durations until testing was performed: (i) natural (28 ± 2 °C, 80% ± 5% RH, ambient CO_2_) and (ii) accelerated carbonation (28 ± 2 °C, 80% ± 5% RH, 10% CO_2_) curing. After the curing period, the samples were exposed to high temperatures at 50, 100, 200, and 300 °C for 2 h to verify the transformation path of the different HMCs. The samples are listed and described in Table 3. The sample labeling follows the format of M age-CO_2_ exposure-temperature. “A” refers to the air content of CO_2_.

After curing, the samples were broken into small pieces and vacuum dried. The outer surfaces were selected and milled for XPS analysis. Powder samples were then fixed in a holder with an adhesive-backed copper tape, and their chemical composition was verified with a Thermo Fisher Scientific Theta Probe system (Thermo Fisher Scientific, Waltham, MA, USA) equipped with a monochromatic Al-Kα (1486.6 eV) X-ray source and a hemispherical electron energy analyzer (Thermo Fisher Scientific). During spectrum acquisition, the pass energy of the analyzer was set to 150 eV for the survey spectra and 40 eV for the individual Mg2p, C1s, and O1s core-level spectra. With this spectrum acquisition set-up, the full width at half maximum (FWHM) of the Ag 3d_5/2_ photoelectron peak was 0.5 eV. The base pressure of the system is below 10^−9^ mbar. A low-energy electron flood gun was used to compensate the positive charge induced by the emission of photoelectrons from an insulating sample. The spectra were curve-fitted using the CasaXPS and OriginPro software applications. In all cases, the secondary electron background was subtracted using the Shirley function. To eliminate any positive charge-induced binding energy shift, the XPS spectra obtained are referenced to the C1s peak from the adventitious carbon at 284.8 eV.

Data on C1s peaks are not presented here; however, they are mentioned and considered in the analysis.

## 3. Results and Discussion

### 3.1. Samples Exposed to High Temperatures

**Figure 1:**
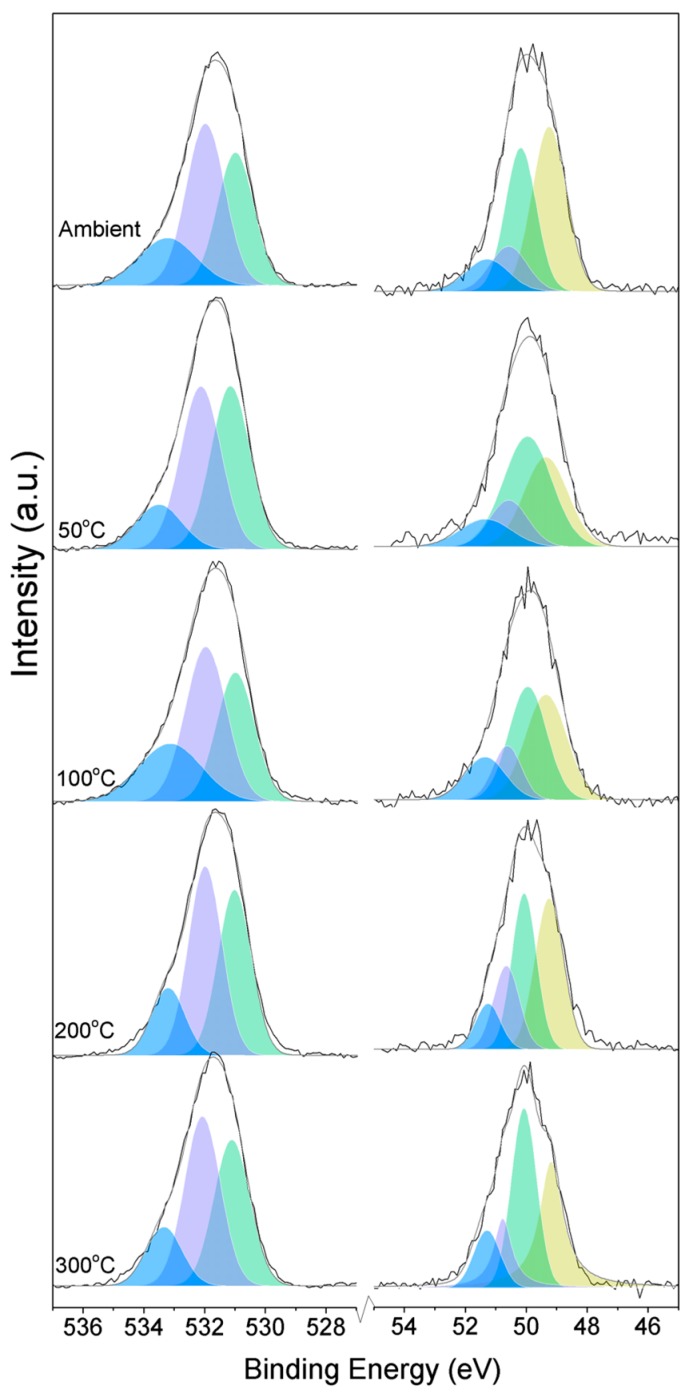
O1s (**left**) and Mg2p (**right**) peaks for samples exposed to different temperatures.

Three O1s peaks are easily distinguished in the XPS spectra of all of the samples (Figure 1). From the literature, it is known that the peaks related to magnesium oxides have BEs at approximately 530.0–531.0 eV, magnesium hydrates (MH) have BEs at approximately 530.0–533.2 eV, and magnesium carbonates present peaks in the range of 533.2–533.5 eV [19,20,21,22,23,24,25]. Due to the enthalpy impact from the BE of its three water molecules, nesquehonite is the most stable phase at pCO_2_ = pH_2_O = 1 bar at up to 46 °C. As the temperature increases, magnesite becomes the most stable phase, followed by MgO [4].

**Figure 2:**
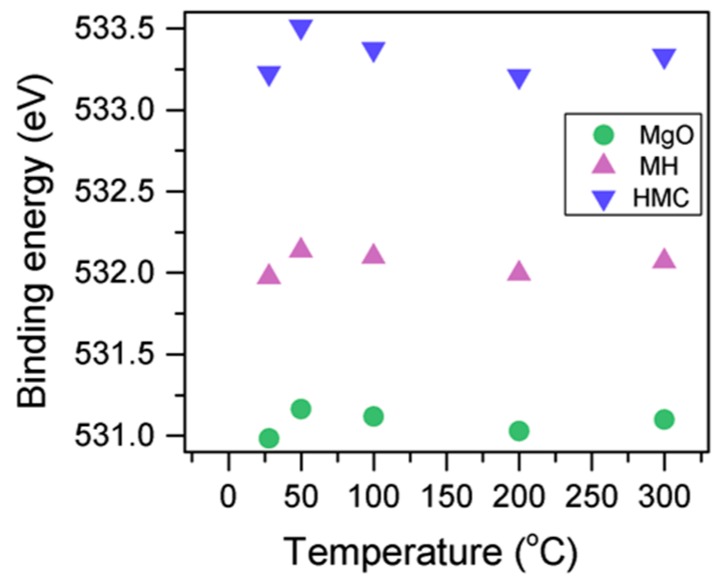
Evolution of the O1s peak position as the temperature increases.

**Figure 3:**
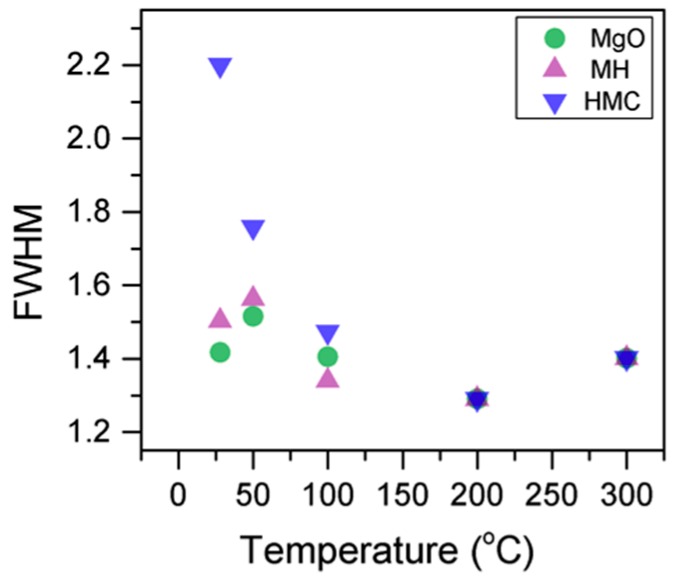
Evolution of the O1s FWHM as the temperature increases.

**Figure 4:**
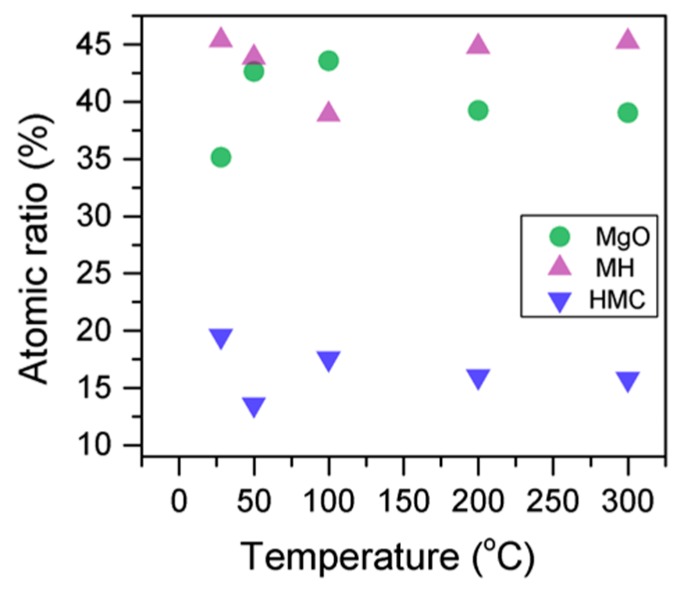
Percentage of different oxygen components for samples subjected to different temperatures.

Here, changes are observed when the samples are subjected to an increase in the temperature; the following describes the phase changes: the O1s peak at higher temperature, related to the magnesium carbonates, shifts initially to a higher BE (533.2 to 533.5 eV) (Figure 2, Table 4), which can be attributed to the transformation of nesquehonite into hydromagnesite, followed by a gradual decrease in the BE as the temperature increases further, which could be due to the formation of magnesite by the dehydration of the sample. The atomic ratio of the carbonates fluctuates; however, as expected, no large changes are observed since the decarbonation of the samples only starts at higher temperatures (Figure 3, Table 4), while the decrease in the FWHM confirms the expected changes in the oxygen bonding (Figure 4, Table 4).

As previously emphasized, magnesite and brucite are the only stable phases in the MgO–CO_2_–H_2_O system, and the increase of the temperature leads to the formation of less hydrated phases. At temperatures above 10 °C, lansfordite decomposes into nesquehonite [3,6]; therefore, lansfordite is not expected to be observed here. Due to the loss of water from the system, which results in the reduction of water activity, above 50 °C, nesquehonite is converted into hydromagnesite [6,26]. However, intermediate phases may be present. Between 100 and 200 °C, the dehydration of hydromagnesite can be observed, and the formation of amorphous magnesite occurs at 126 °C. Above 315 °C, the decarbonation process starts, which leads to the formation of crystalline magnesite. The formation of periclase, which is the only phase observed at higher temperatures, can be observed at temperatures as low as 330 °C [27].

Under ambient temperature and CO_2_ concentrations, the formation of artinite and lansfordite (MgCO_3_·5H_2_O) is expected. The formation of artinite requires less CO_2_ than the amount present under ambient conditions (8–40 × 10^−4^ vs. 0.04%) [2]. An increase in the temperature results in the transformation of these HMCs into nesquehonite [7,12], which can also form under ambient temperature and CO_2_ concentrations [9,28,29,30]. Other studies have shown that nesquehonite forms at a temperature and CO_2_ pressure of ~25–55 °C and 1 bar, respectively [29,31,32]. Nesquehonite is stable at temperatures as high as 55–100 °C, after which it loses its water of crystallization [17,33,34,35]. The presence of hydromagnesite at lower temperatures and ambient CO_2_ concentrations has also been reported [30]. The dehydration of hydromagnesite starts at 135 °C, followed by dehydroxylation at approximately 184 °C [36]. Other studies have shown that hydromagnesite is stable up to 300 °C [15]. Dypingite, which exists under elevated temperatures, is usually observed as an intermediate phase between nesquehonite and hydromagnesite [9,37].

There are contradictions in the literature with regards to the Mg2p binding energy (BE) of different magnesium components, but the peak with the lowest BE is associated with metallic Mg [21,22,38,39], or single-crystal 0001 Mg [40]. At higher BEs, several authors associate MgO with the peak at approximately 50.3–50.8 eV [20,39,40,41], while Mg(OH)_2_ is represented by the peaks at approximately 49.5 [42] to 51.5 eV [19,22]. Finally, MgCO_3_ is present in peaks from 50.4 [43], through intermediate values [19,20], and up to 54.0 eV [44]. Newberg et al. [45] presented a detailed study on the reaction of MgO thin films with water in ambient temperature using XPS, which demonstrated how molecular and dissociative water react with the MgO to form the hydrated products.

The Mg2p level is close to the valence band and, therefore, it is easy to observe changes in the valence band and to identify the valence state for this element. In this case, a shift to higher binding energies occurs when a valence electron is lost because the bonds become stronger, i.e., anions have lower BEs than atoms. Therefore, for the Mg2p peaks, it is inferred that the peak at a higher BE is related to MgCO_3_ or to dioxygen species, such as magnesium peroxide, with Mg in the form of (2+) [44].

As expected, the samples present magnesium with different coordinations, which means that they form different components. All samples present four components for Mg2p: at BEs of 49.2 eV (associated with metallic Mg, which can be a residue from the precursor during the synthesis), 50.1 eV (that may represent MgO), and 50.5 eV (related to the Mg-O/OH species [22], which can be MgOH [22,38,40,41,46]). Mg2p peaks present at low BE, such as the peak at 49.2 eV, are normally associated with pure, metallic Mg. The specimens present an additional peak at approximately 51.4 eV, which is associated with crystalline Mg–OH [22], i.e., Mg(OH)_2_, or to MgCO_3_.

When the samples are subjected to high temperatures, it is observed that the Mg2p peak related to metallic Mg shifts by 0.2 eV to a higher BE but soon fluctuates down to the initial BE. Simultaneously, the peak related to MgO shifts to a lower BE (50.2 to 50.0 eV), and this increases again when the samples are subjected to temperatures higher than 100 degrees; however, the BE is still lower than the initial BE by 0.1 eV, and the Mg(OH)_2_ peak shows a 0.2 eV shift to a higher BE only when the temperature is at 300 °C. The peak at a higher BE, related to the magnesium carbonates, does not exhibit any significant change in terms of BE. In the literature, samples heated to high temperatures, however, present abnormalities on the adventitious carbon peak, which end up inducing errors on the BE of the magnesium peak [47,48,49]. These studies correct the charging effect by using alternative peaks, usually the O1s peak, when it originates only from the native oxide; however, this cannot be ensured here. Therefore, the Mg2p BE here cannot be considered as stand-alone information on the chemical state of the elements, and it is analyzed together with the concentration, FWHM, and the other components present in the sample.

**Figure 5:**
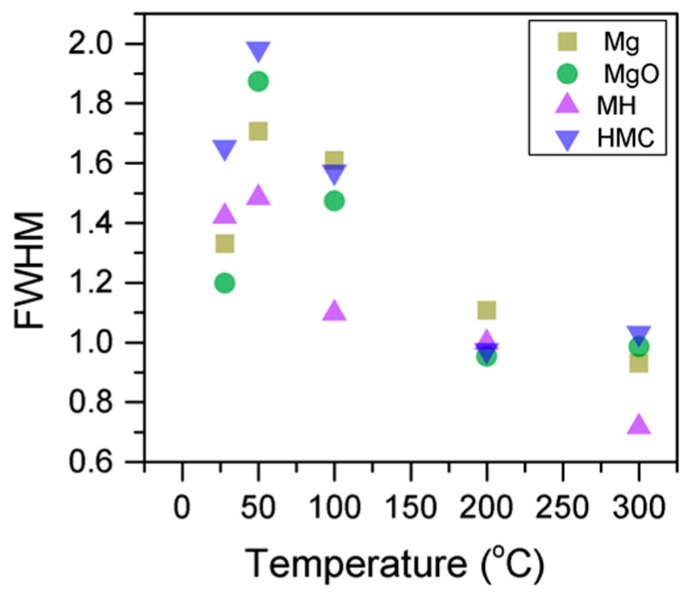
Evolution of the Mg2p FWHM as the temperature increases.

Here, the FWHM reduces significantly for the all the elements, which indicates changes in the chemical state; in particular, in magnesium hydrates, where the decrease is more significant, the reduction in the FWHM indicates the decomposition of these components (Figure 5). At the same time, the amount of MgCO_3_ in the samples fluctuates as the temperature increases, as there is not much decarbonation within this range; moreover, the other components do not provide significant information on the atomic ratio.

There is a C1s signal at approximately 290 eV (not presented here) due to carbon-oxygen bonding, which may be O–C=O. It is known that the carbon atoms in the magnesite phase are threefold-coordinated carbon atoms with triangular CO^2−^ ions with sp^2^ bonding [50], which is likely observed here. Feliu et al. [21] in their experiments involving the exposure of pure Mg to an ambient environment with high humidity, also describe observing hydration and carbonation and associating peaks at approximately 290.3–290.8 eV to magnesium carbonates. Here, all of the samples that are either subjected or not subjected to high temperatures gradually present the same peak at approximately 289.8 eV, which is related to magnesium carbonates, and no significant differences in the FWHM value are observed.

### 3.2. Effect of Exposure to Air or CO_2_

**Figure 6:**
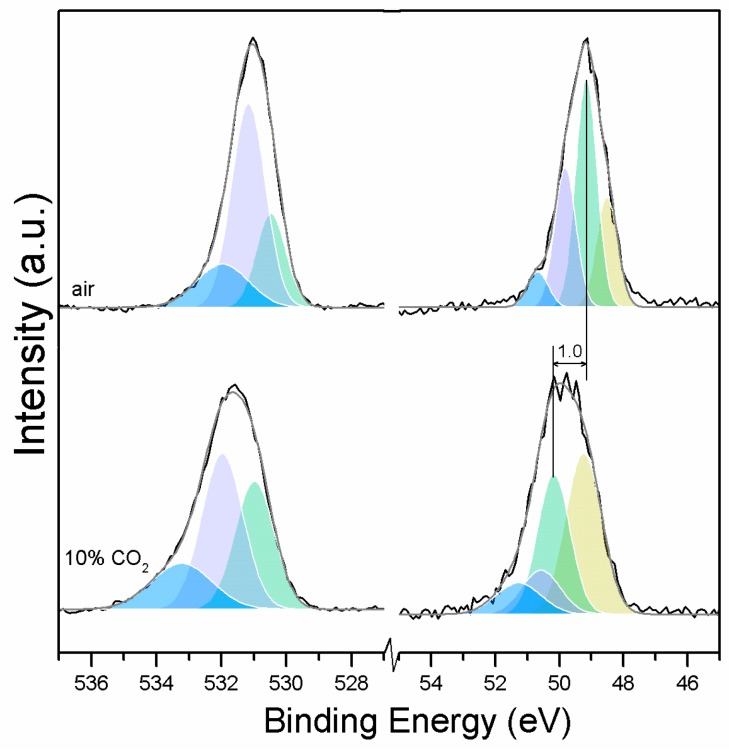
O1s (**left**) and Mg2p (**right**) peaks for samples exposed to air and 10% CO_2_.

**Figure 7:**
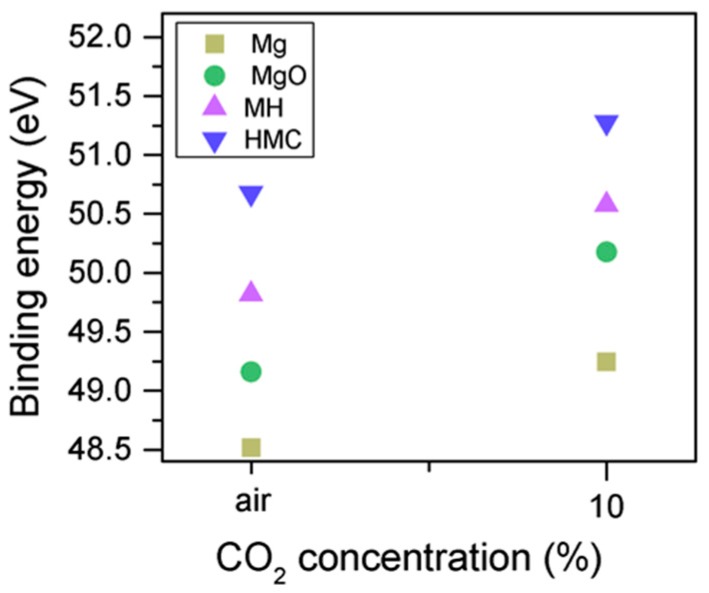
Mg2p BE for samples exposed to air and CO_2_ for seven days.

Clear differences are observed between a sample of MgO cement with a w/c ratio of 0.55 exposed for seven days to either air or CO_2_. Firstly, and most importantly, only a very small Mg2p peak at high energies, related to carbonates, is present for the sample not exposed to CO_2_ (Figure 6 and Figure 7). This confirms that there is minimal formation of magnesium carbonate hydrates when the sample is not exposed to a high concentration of CO_2_ in such early ages, even at the surface. Secondly, the binding energies increase for all the Mg2p peaks as the exposure time increases. This is related to the formation of hydrated and carbonated species. The peak at a lower energy, which is related to metallic Mg, has its BE increased by 0.7 eV, while the initial BE for the MgO peak rises by 1.0 eV (the arrow in Figure 7), approaching the energies of Mg(OH)_2_, and clearly showing the reaction with water, which leads to the formation of hydrated species. Finally, the initial BE for Mg(OH)_2_ is increased by 0.8 eV in the direction of the BE of the carbonated species (Figure 7).

**Figure 8:**
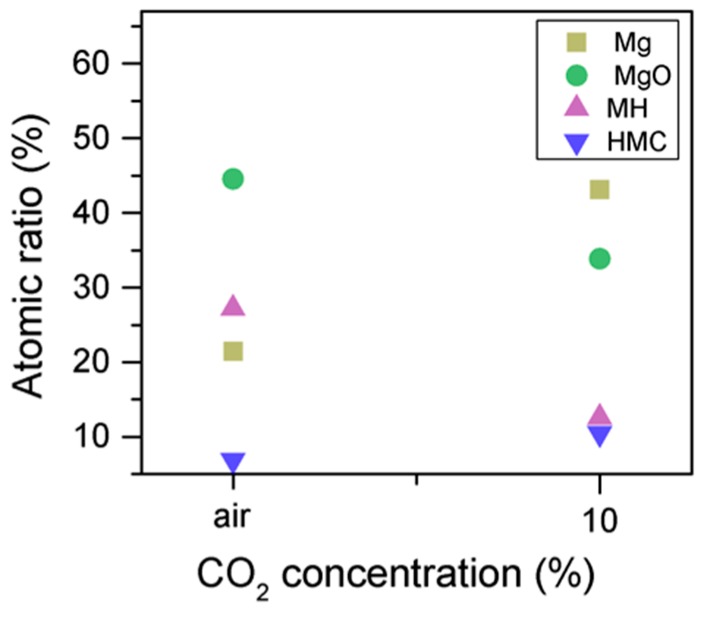
Mg2p concentration for samples exposed to air and CO_2_ for seven days.

Similarly, the FWHM for all Mg peaks increases upon the exposure to CO_2_ (Figure 6, Table 5). However, even though the concentration of magnesium in MgO and Mg(OH)_2_ seems to decrease when the sample is exposed to CO_2_, the metallic Mg peak concentration seem to increase along with the increase in the concentration of carbonates (Figure 8). This may be due to differences between the samples or even different scanning locations in the sample, where there may be higher concentrations of pure Mg from the precursor.

**Figure 9:**
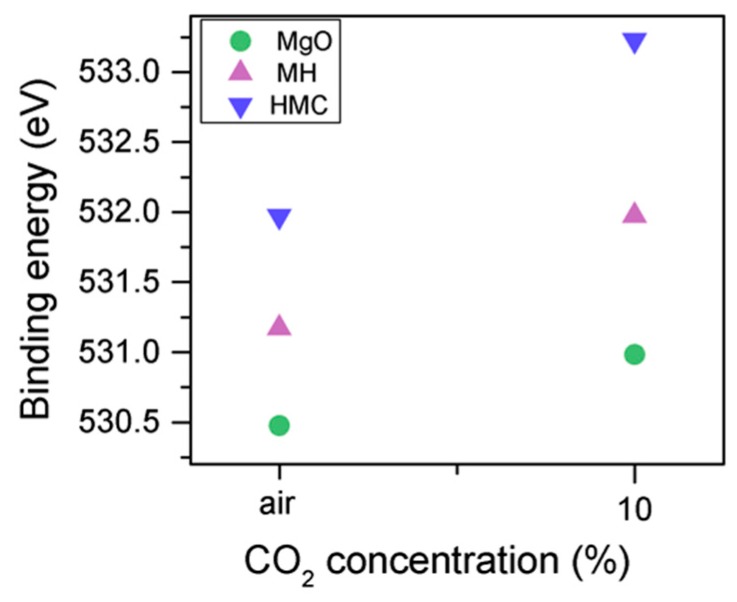
O1s BE for samples exposed to air and CO_2_ for seven days.

**Figure 10:**
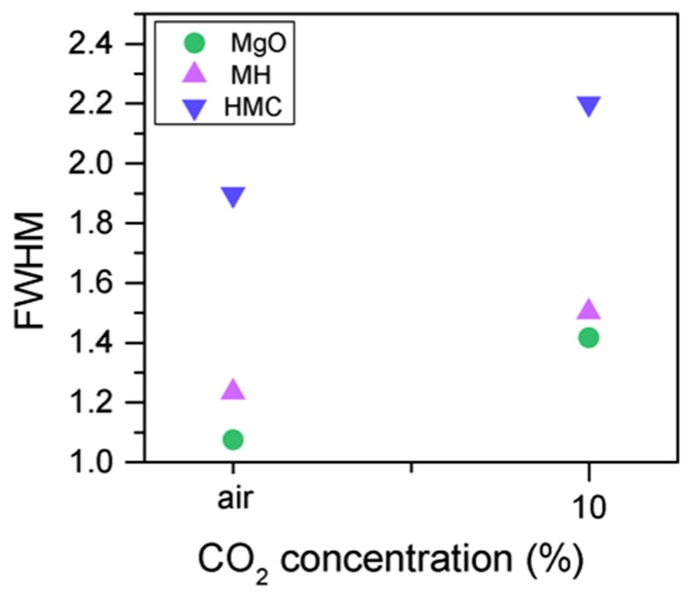
O1s FWHM for samples exposed to air and CO_2_ for seven days.

The C1s peak (not presented here) indicates differences when the sample is exposed to CO_2_ instead of air: an increase of the BE by 0.8 eV is observed (from 289.0 to 289.8 eV), which is accompanied with a decrease of the FWHM by 0.8 eV. On the other hand, the O1s peaks shift by 0.2 eV to higher BEs, which also indicates the formation of magnesium carbonates; the magnesium hydrates peak shifts from 531.8 to 532.0 eV (Figure 9), while the concentration of carbonates increases significantly (Table 5), with a large increase in the FWHM of the carbonated species (Figure 10).

### 3.3. Effect of the Length of CO_2_ Exposure

**Figure 11:**
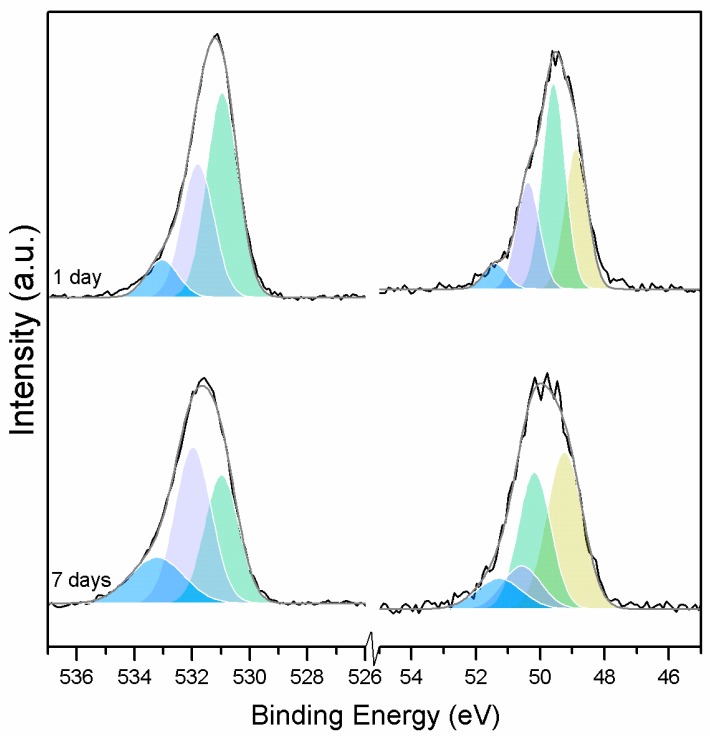
O1s (**left**) and Mg2p (**right**) peaks for samples exposed to 10% CO_2_ for one and seven days.

**Figure 12:**
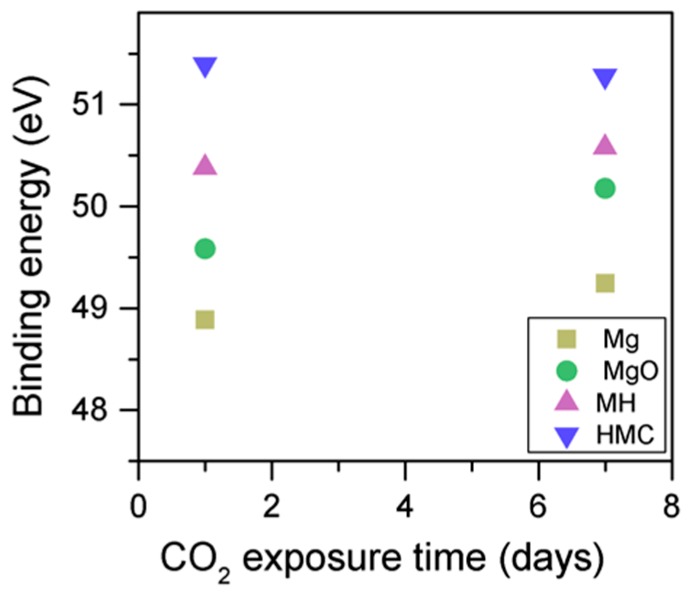
Mg2p BE for samples exposed to 10% CO_2_ for one and seven days.

When samples exposed to CO_2_ for one and seven days are compared, a clear change is observed in the Mg2p peaks related to metallic Mg, periclase, and magnesium hydrates (Figure 11); a shift to a higher BE is observed, which indicates the formation of hydrates and carbonates. The metallic Mg peak shifts by 0.3 eV towards a BE closer to MgO. The BE of the MgO peak increases by 0.6 eV towards the BE of the magnesium hydrates, and the peak for the magnesium hydrates increases by 0.2 eV in the direction of the BE of the magnesium carbonates. However, no significant changes in BE are observed for the peak at higher BE, which is related to the carbonates (Figure 12).

**Figure 13:**
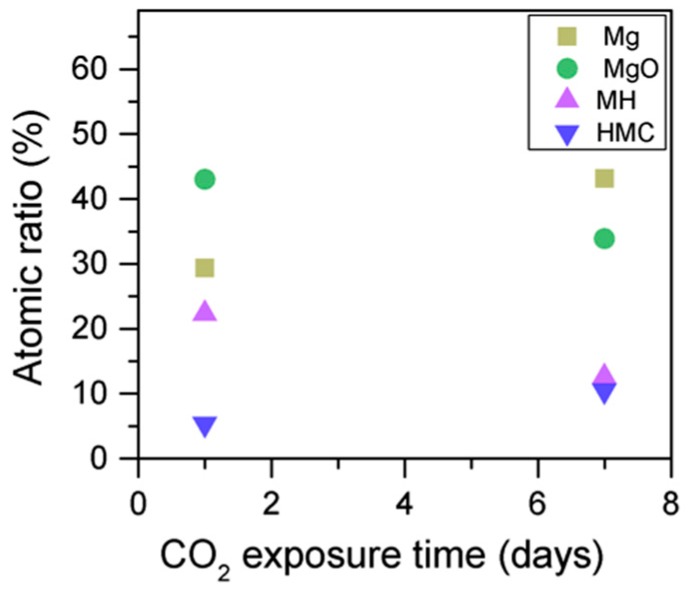
Mg2p concentration for samples exposed to 10% CO_2_ for one and seven days.

Simultaneously, the decrease in the concentration of periclase and the hydrated components, along with an increase in the concentration of carbonates, again confirms the formation of these elements in the samples as the exposure time increases (Figure 13). Similarly, the FWHM values for all components increase after the exposure to CO_2_; however, the peak related to the carbonates presents significantly larger changes, almost duplicating its FWHM values (Table 6).

**Figure 14:**
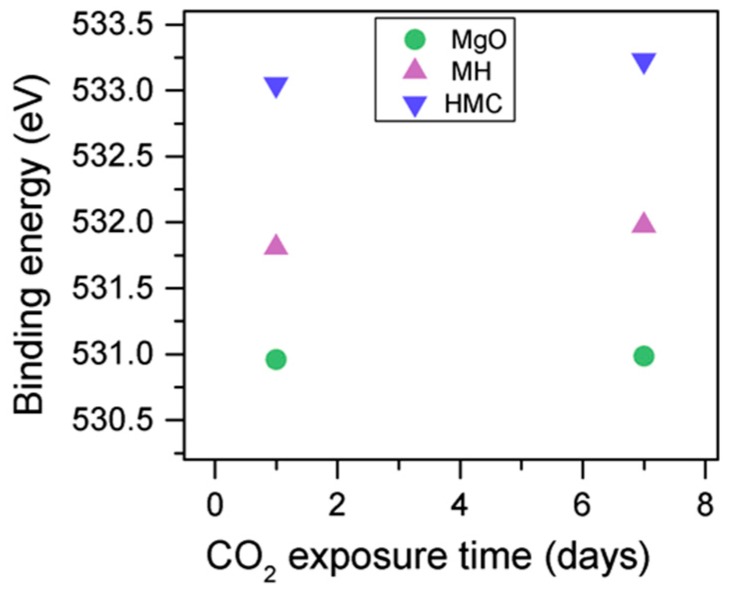
O1s BE for samples exposed to 10% CO_2_ for one and seven days.

**Figure 15:**
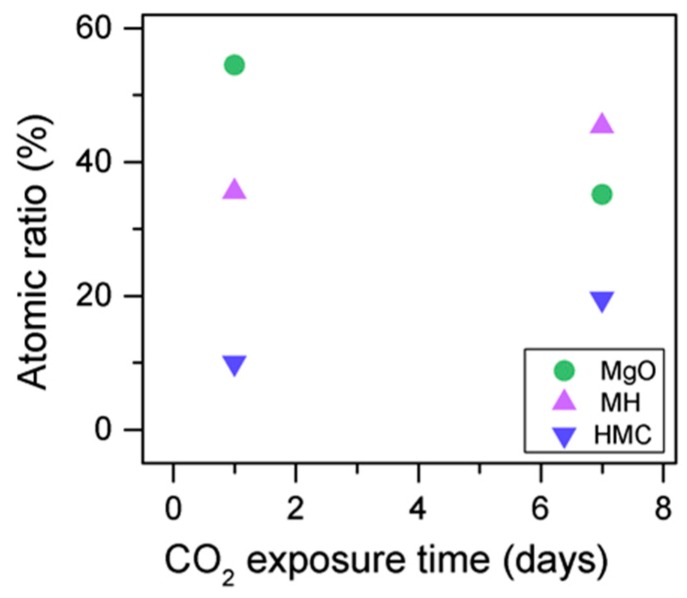
O1s atomic concentration for samples exposed to 10% CO_2_ for one and seven days.

**Figure 16:**
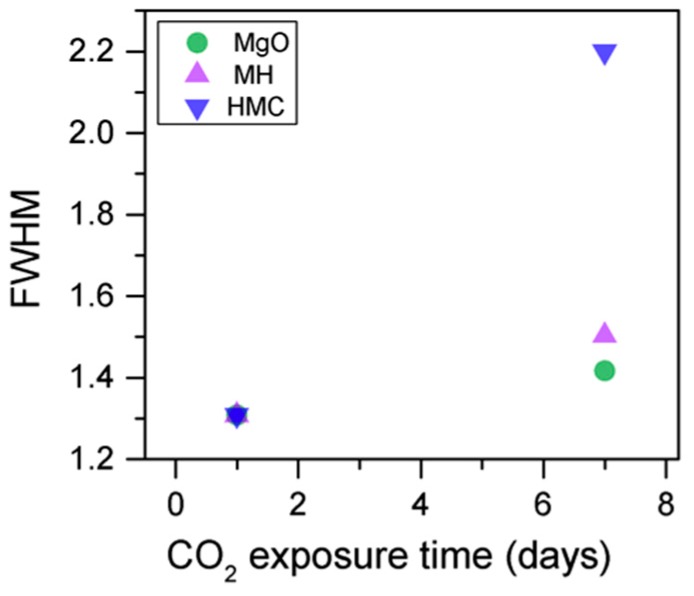
O1s FWHM for samples exposed to 10% CO_2_ for one and seven days.

The C1s peaks (not presented here) show a slight 0.1 eV shift to a higher BE for a longer exposure time, which is accompanied by a decrease of the FWHM by 0.2 eV. The O1s peaks shift to higher BE when the sample is exposed for a longer time for both the magnesium carbonates (533.0–533.2 eV) and hydrates (531.8–532.0 eV), indicating the formation of carbonates, while the peak for the oxides does not change (Figure 14). In addition, the concentration of carbonates is observed to increase together with an increase in the concentration of magnesium hydrates and a decrease in the concentration of periclase (Figure 15). Finally, Figure 16 presents the variation on FWHM.

## 4. Conclusions

This study presented the ability of reactive MgO cement-based formulations to carbonate and established a link between the formation of various carbonate phases and specific controlled parameters. The influence of three parameters (i.e., the temperature, CO_2_ concentration and exposure time) on the progress of carbonation and CO_2_ sequestration potential of MgO cement blends was identified by evaluating the chemical changes in the elemental composition of the formed carbonate phases through the use of surface characterization techniques.

The surface changes upon the hydration and carbonation of the studied MgO cement systems provided insight into the formation, chemical composition, and thermal decomposition of the various carbonate phases over time. The changes in BE indicated the formation of different components and the transformation of the components from one form to another. Some of the key findings of this study are listed as follows:

- Increases in temperature led to the transformation of nesquehonite into hydromagnesite, as indicated by the changes in the BE of the O1s peak. This was eventually followed by the conversion of hydromagnesite into magnesite. The atomic ratio of the carbonates fluctuated without significant changes, as decarbonation is expected to only start at higher temperatures. Decreases in the FWHM indicated changes in the oxygen bonding. Four components were observed for Mg2p in all samples, which are related to metallic Mg (which can be trapped inside particles from the precursor), MgO, MH and HMCs. Their BEs could not be evaluated alone due to abnormalities in the adventitious carbon peak observed in the literature for samples heated to elevated temperatures, and instead, the BEs were analyzed together with the concentration, FWHM, and the other components present in the sample.
- Increases in the BE of C1s and O1s as the CO_2_ concentration increased indicated the formation of carbonate phases. At high energies, the Mg2p peak (related to the carbonates) was extremely small for the sample exposed to only air, which demonstrated the effect of the carbon concentration on the formation of HMCs. Agreements between the C1s and O1s peaks were observed via the increases in BE with the concentration (from air to 10% CO_2_), while the magnesium hydrates peak shifts and the amount of carbonates increase significantly, together with an increase in the FWHM of the carbonated species.
- An increase in the CO_2_ exposure time from one to seven days was demonstrated via shifts of all components to higher BEs. This was accompanied with the decrease of the concentrations of periclase and the hydrated components and the increase in the concentration of carbonates, which indicated the continuous formation of hydrate and carbonate phases for longer exposure times.

The outcomes of this study advanced the understanding of how chemical changes that occur in the surface of MgO cement systems under three controlled parameters (i.e., temperature, CO_2_ concentration, and exposure time) affect the formation and stability of various carbonate phases within the MgO–CO_2_–H_2_O system. The results obtained shed light on the HMC formation patterns and their decomposition within MgO formulations that are subjected to different conditions. A more detailed identification of relevant intermediate phases can be further achieved via the use of more powerful analysis techniques to overcome the limitations from the complex chemistry of Mg-based phases and provide a complete evaluation of the transformation patterns.

## Figures and Tables

**Table 1:** Compounds formed in the MgO–CO_2_–H_2_O system [1,2,3,4,5].

Group	Number of Mg Ions	Compound	Chemical Formula
		Brucite	Mg(OH)_2_
		Magnesite	MgCO_3_
Group I	1	Barringtonite	MgCO_3_·2H_2_O
		Nesquehonite	MgCO_3_·3H_2_O
		Lansfordite	MgCO_3_·5H_2_O
Group II	2	Pokrovskite	Mg_2_(CO_3_)(OH)_2_·0.5H_2_O
		Artinite	Mg_2_(CO_3_)(OH)_2_·3H_2_O
Group III	5	Hydromagnesite	Mg_5_(CO_3_)_4_(OH)_2_·4H_2_O
		Dypingite	Mg_5_(CO_3_)_4_(OH)_2_·5H_2_O
		Giorgiosite	Mg_5_(CO_3_)_4_(OH)_2_·5–6H_2_O
Group IV	7	Shelkovite	Mg_7_(CO_3_)_5_(OH)_4_·24H_2_O

**Table 2:** Chemical composition and physical properties of MgO.

	Chemical Composition (%)							Physical Properties	
	MgO	SiO_2_	CaO	R_2_O_3_	K_2_O	Na_2_O	LOI	Specific Gravity (g/cm^3^)	Specific Surface Area (m^2^/g)
RMC	>91.5	2.0	1.6	1.0	-	-	4.0	3.0	16.3

**Table 3:** Samples analyzed under this study.

Sample	Age (Days)	CO_2_ Exposure	Temperature (°C)
M7-10-50	7	10%	50
M7-10-100	7	10%	100
M7-10-200	7	10%	200
M7-10-300	7	10%	300
M1-10-28	1	10%	28
M7-10-28	7	10%	28
M7-A-28	7	air	28

**Table 4:** Peak information for Mg2p and O1s at different temperatures.

	Mg2p			O1s		
Temperature (°C) Sample ID	Peak Position (eV)	Concentration (%)	FWHM	Peak Position (eV)	Concentration (%)	FWHM
0 M7-10-28	49.2	43.1	1.33	531.0	35.1	1.42
	50.2	33.9	1.20	532.0	45.3	1.50
	50.6	12.6	1.42	533.2	19.5	2.20
	51.3	10.4	1.65	-	-	-
50 M7-10-50	49.4	31.6	1.71	531.2	42.6	1.51
	50.0	42.7	1.87	532.1	43.8	1.56
	50.6	14.3	1.48	533.5	13.5	1.76
	51.3	11.4	1.98	-	-	-
100 M7-10-100	49.3	36.6	1.61	531.1	43.6	1.40
	49.9	36.0	1.47	532.1	38.9	1.34
	50.6	12.8	1.10	533.4	17.5	1.47
	51.3	14.6	1.57	-	-	-
200 M7-10-200	49.2	37.7	1.11	531.0	39.2	1.29
	50.1	33.5	0.95	532.0	44.8	1.29
	50.6	18.8	1.00	533.2	16.0	1.29
	51.2	10.0	0.97	-	-	-
300 M7-10-300	49.2	35.1	0.93	531.1	39.0	1.40
	50.1	37.4	0.99	532.1	45.2	1.40
	50.8	15.0	0.72	533.3	15.7	1.40
	51.3	12.5	1.03	-	-	-

**Table 5:** Mg2p and O1s peak information for samples exposed to air and 10% CO_2_.

	Mg2p			O1s		
CO_2_ Concentration Sample ID	Peak Position (eV)	Concentration (%)	FWHM	Peak Position (eV)	Concentration (%)	FWHM
Air M7-A-28	48.5	21.4	0.8	530.5	23.2	1.1
	49.2	44.5	0.8	531.2	57.9	1.2
	49.8	27.2	0.8	532.0	18.9	1.9
	50.7	6.9	0.8	-	-	-
10% CO_2_ M7-10-28	49.2	43.1	1.3	531.0	35.1	1.4
	50.2	33.9	1.2	532.0	45.3	1.5
	50.6	12.6	1.4	533.2	19.5	2.2
	51.3	10.4	1.7	-	-	-

**Table 6:** Mg2p peak information for samples exposed to 10% CO_2_ for one and seven days.

	Mg2p			O1s		
Exposure Time (Days) Sample ID	Concentration %	Peak Position (eV)	FWHM	Concentration %	Peak Position (eV)	FWHM
1 M1-10-28	29.4	48.9	0.8	54.5	531.0	1.3
	43.0	49.6	0.8	35.5	531.8	1.3
	22.3	50.4	0.8	10.0	533.0	1.3
	5.3	51.4	0.8			
7 M7-10-28	43.1	49.2	1.3	35.1	531.0	1.4
	33.9	50.2	1.2	45.3	532.0	1.5
	12.6	50.6	1.4	19.5	533.2	2.2
	10.4	51.3	1.7			
